# Draft Genome Assemblies of Xylose-Utilizing *Candida tropicalis* and *Candida boidinii* with Potential Application in Biochemical and Biofuel Production

**DOI:** 10.1128/genomeA.01594-17

**Published:** 2018-02-15

**Authors:** Abhishek Somani, Daniel Smith, Matthew Hegarty, Narcis Fernandez-Fuentes, Sreenivas R. Ravella, Joe A. Gallagher, David N. Bryant

**Affiliations:** a Institute of Biological, Environmental & Rural Sciences, Aberystwyth University, Aberystwyth, United Kingdom

## Abstract

Non-*albicans Candida* species are growing in prominence in industrial biotechnology due to their ability to utilize hemicellulose. Here, we present the draft genome sequences of an inhibitor-tolerant *Candida tropicalis* strain (Y6604) and *Candida boidinii* NCAIM Y01308^T^.

## GENOME ANNOUNCEMENT

Manufacturing higher-value commodities from hemicellulosic sugars (e.g., xylose) is crucial for environmental and bioeconomic sustainability of lignocellulosic biorefineries. *Candida tropicalis* has been widely investigated in the bioconversion of xylose into the higher-value sweetener xylitol and/or into bioethanol (1, 2), while the methylotroph *Candida boidinii* is well established for heterologous gene expression and enzyme/biochemical production (3, 4). In this study, we report the draft genome sequences of an environmentally derived inhibitor-tolerant *C. tropicalis* isolate (Y6604) and *C. boidinii* NCAIM Y01308^T^ (NCAIM, Budapest, Hungary). Strain Y6604 has high tolerance to lignocellulose-derived inhibitors (up to 3 g/liter furfural and 4 g/liter 5-hydroxymethylfurfural), and metabolically engineered variants have improved xylose-to-xylitol bioconversions in lignocellulosic hydrolysates (data not shown).

Genomic DNA (1 µg) (YeaStar; Zymo Research, USA) was extracted, and Illumina TruSeq libraries were size selected with AMPure beads for an average insert size of ~700 bp. Prior to sequencing on an Illumina HiSeq 2500 platform, paired-end reads were produced for both species, with additional mate pair reads for *C. boidinii* NCAIM Y01308^T^. Overlapping paired-end sequence reads were merged using FLASH (5). The quality control suite FastQC (http://www.bioinformatics.babraham.ac.uk/projects/fastqc/) identified some initial 5′ base bias, low-quality 3′ bases, and Illumina adaptors, which were removed using Trimmomatic (6). NxTrim (7) was used for adaptor trimming of raw *C. boidinii* mate pair sequences. All sequences were error corrected and assembled using SOAPdenovo (8). Following genome masking, coding genes predicted by AUGUSTUS (9) and trained for *C. tropicalis* were submitted to a BLASTp search. Candidate hits (E value, ≤1 × 10^−10^) were assigned names, Gene Ontology (GO) and Kyoto Encyclopaedia of Genes and Genomes (KEGG) annotations using Blast2GO (10).

A total of 51,470,621 paired-end reads for Y6604 gave an assembly length of 14,318,547 bp, with 296× coverage of 563 scaffolds (*N*_50_, 51,027), and 10,177 contigs. Scaffolds with more than 50% “N” calls and <300 coding bases were removed, giving 533 scaffolds and 688 contigs. The combined *C. boidinii* assembly from both paired-end and mate pair reads had high coverage depths (259× and 523×, respectively) and a total length of 19,266,739 bases. Scaffolds with <1,000 coding bases and/or more than 50% N calls were removed, giving 79 scaffolds (*N*_50_, 606,681) and 61 contigs. The overall GC content in Y6604 was 34%, while that in *C. boidinii* was lower (31%). *De novo* annotation with AUGUSTUS yielded 6,772 proteins in Y6604 (8,270 exons and 1,498 introns) and 6,067 proteins (6,951 exons and 884 introns) in *C. boidinii* NCAIM Y01308^T^. Reciprocal best BLAST hits (maximum E value, 1 × 10^−10^) for Y6604 proteins compared with the 6,254 proteins in the reference *C. tropicalis* (strain MYA-3404) assembly (11) identified 5,487 matching proteins, with 1,285 and 767 proteins unique to Y6604 and MYA-3404, respectively. A BUSCO (v3.0.1) (11) comparison between the 1,711 profiles within the order *Saccharomycetales* and the proteins predicted by AUGUSTUS suggested that the strain Y6604 and *C. boidinii* NCAIM Y01308^T^ gene sets were largely complete, with only 6% and 7% (96 and 112 genes, respectively) of the conserved orthologs, respectively, deemed missing.

### Accession number(s).

The *C. tropicalis* strain Y6604 and *C. boidinii* NCAIM Y01308^T^ whole-genome shotgun projects have been deposited in DDBJ/ENA/GenBank under the accession numbers PKKZ00000000 and PKKY00000000, respectively. The versions described in this paper are the first versions.
